# Pharmacology educators as leaders of programmatic assessment for safe prescribing in competency-based medical education

**DOI:** 10.3389/fphar.2026.1895683

**Published:** 2026-07-29

**Authors:** Munder Zagaar, Kelly Karpa, John L. Szarek, Jennifer Koenig, Lindsay Cormier, Janet Mifsud, Katerina Venderova, Carolina Restini

**Affiliations:** 1 Department of Biochemistry and Molecular Pharmacology, School of Medicine, Baylor College of Medicine, Houston, TX, United States; 2 Department of Medical Education, East Tennessee State University, Quillen College of Medicine, Johnson City, TN, United States; 3 Department of Medical Education, Geisinger College of Health Sciences School of Medicine, Scranton, PA, United States; 4 School of Medicine, University of Nottingham, Nottingham, United Kingdom; 5 Department of Pharmacology and Nutritional Sciences, Lindsey Cormier, University of Kentucky, Lexington, KY, United States; 6 Department of Clinical Pharmacology and Therapeutics, Faculty of Medicine and Surgery, University of Malta, Msida, Malta; 7 Department of Biomedical Science, Kaiser Permanente Bernard J. Tyson School of Medicine, Pasadena, CA, United States; 8 Department of Pharmacology and Toxicology, College of Osteopathic Medicine, Michigan State University, East Lansing, MI, United States

**Keywords:** clinical decision-making, competency-based medical education, entrustable professional activity, integrated curriculum, medication errors, pharmacology education, pharmacology thread, prescribing competence

## Abstract

Unsafe prescribing remains a persistent source of preventable patient harm despite extensive pharmacology instruction in medical curricula. This gap likely reflects not a lack of content in the curricula, but a failure of structured curriculum integration, accountability, and longitudinal assessment. We argue that competency-based medical education (CBME), particularly through Entrustable Professional Activity 4 (EPA 4), requires a fundamental redefinition of the pharmacology educator’s role - from content expert to leader of programmatic assessment for prescribing competence. We propose a model of pharmacology thread leadership grounded in cognitive integration (spiraling, spacing, and interleaving), governance authority, and fidelity metrics to ensure consistency between what is taught, assessed, and practiced. This model aligns pharmacological reasoning with clinical decision-making through multi-method programmatic assessment, which may include written assessments, prescribing Objective Structured Clinical Examinations, workplace-based observations, and simulation. We further outline the structural enablers necessary for implementation, including faculty development, equity-focused design, and institutional support. While centered on pharmacology, this framework provides a transferable template for other foundational sciences seeking to achieve measurable impact on clinical competence. This paper reinforces the key role pharmacology educators have in integrated medical curricula by claiming explicit leadership in the EPA-aligned system of assessment, advancing multi-institutional research on prescribing outcomes, explicitly highlighting pharmacology as a discipline essential to well-supported decisions about readiness for practice, in other words, entrustment decisions, and patient safety.

## 1 Introduction: prescribing competence requires ownership, not exposure

Prescribing is one of the most consequential activities expected of graduating medical students, yet it remains a persistent source of preventable harm worldwide (Wiernik, 2015). Errors in medication selection, dosing, monitoring, and communication persist despite extensive curricular attention to pharmacology and therapeutics (Heaton et al., 2008; Maxwell et al., 2015). This disconnect reflects a fundamental limitation in current educational models: prescribing competence is treated as a distributed outcome without clear ownership, longitudinal structure, or accountability for assessment (Brinkman et al., 2017; 2018).

Competency-based medical education (CBME) has sought to address this gap by defining observable professional activities, such as prescribing and medication management under supervision, that inform entrustment decisions, for example, Entrustable Professional Activity 4 (EPA-4) in the USA medical schools (Ten Cate, 2013; Mejicano et al., 2017). However, implementation remains uneven, and in many programs, prescribing competence is inferred from fragmented assessments rather than demonstrated through integrated, longitudinal evidence (Frank et al., 2010; Association of American Medical Colleges, 2014; Englander et al., 2016; Gruppen et al., 2016).

We contend that this gap is not primarily curricular, but structural. Pharmacology, the discipline most directly responsible for developing the mechanistic reasoning essential for safe prescribing, remains largely confined to content delivery rather than being positioned as a leader in assessment systems (Bloch et al., 2025). As a result, pharmacological reasoning is inconsistently integrated into assessments of clinical decision-making and insufficiently represented in entrustment decisions (Association of American Medical Colleges, 2014; Wiernik, 2015; Brinkman et al., 2017; 2018; Fasinu and Wilborn, 2024).

This challenge is not unique to a single educational system. International frameworks similarly emphasize prescribing as a core competency requiring structured oversight. For example, the UK General Medical Council outlines expectations for safe prescribing and medication management across training, while European Union directives mandating cross-border recognition of prescriptions underscore the need for harmonized and benchmarked medical education across jurisdictions (European Parliament and the Council of the European Union, 2011; General Medical Council, 2021). Together, these initiatives reinforce the idea that prescribing competence must be developed longitudinally and assessed continuously within coherent educational systems, rather than assumed through exposure alone.

For these reasons, prescribing must be seen as not solely a clinical task but grounded in explicit pharmacological reasoning. Understanding how drugs work provides the basis for safe and effective decision-making, including adapting therapy when standard approaches do not fit, managing adverse effects, and accounting for comorbidity, organ dysfunction, and drug interactions (Fasinu and Wilborn, 2024; Guilding et al., 2026). Without these conceptual foundations, prescribing becomes overly dependent on routine patterns and may fail when clinical judgment under uncertainty is required (Norman, 2009).

Several recent publications have advanced the field of pharmacology education by defining core disciplinary concepts, updating prescribing curricula, evaluating prescribing competence, and identifying opportunities to strengthen pharmacology teaching within integrated medical programs (Fasinu and Wilborn, 2024; Guilding et al., 2024; 2026; McLachlan et al., 2025; Lonsdale et al., 2026). Collectively, these contributions have clarified what learners should know, what prescribing skills should be assessed, and why pharmacology remains essential to safe patient care. However, a persistent gap remains regarding who is responsible for ensuring that pharmacological reasoning is developed, assessed, and monitored longitudinally across the curriculum. To address this gap, we propose a governance model that explicitly assigns responsibility, authority, and accountability for the longitudinal assessment of prescribing competence to pharmacology thread leadership. Rather than introducing new prescribing competencies, assessment instruments, or pharmacology learning outcomes, our model focuses on the organizational structures needed to sustain curricular coherence, coordinate assessment systems, and support entrustment decisions over time. This perspective, therefore, extends existing discussions of curriculum design and assessment by emphasizing that governance mechanisms are a responsibility of pharmacology educators, enabling the translation of educational principles into sustained program-level practice.

Our proposed model draws upon complementary educational theories and implementation frameworks, including transfer of learning, situated learning, backward design, competency-based medical education, and programmatic assessment, to position pharmacology educators as leaders of longitudinal prescribing assessment.

We argue that pharmacology educators must move beyond episodic, piecemeal content delivery toward leading integrated, programmatic assessment systems that capture the development of prescribing competence over time. The discussion first considers how pharmacological reasoning should be organized and reinforced across contexts, and then addresses the governance structures, assessment architectures, and evaluative practices required to well-supported entrustment decisions.

This perspective advances a central claim: pharmacology educators must assume program-level leadership in programmatic assessment of prescribing competence to ensure safe, consistent, and equitable outcomes. We propose a framework that integrates cognitive science, governance structures, and assessment architecture to support this role. Thus, the novelty of the proposed model lies not in the individual educational components themselves, but in the assignment of responsibility, authority, and accountability for coordinating them within a single governance structure.

## 2 Cognitive integration as the foundation of prescribing competence

In many integrated curricula, pharmacology is distributed across organ systems with the assumption that proximity leads to integration (Quesnelle et al., 2021; Fasinu and Wilborn, 2024). In practice, learners with developing knowledge of pharmacology and limited metacognitive awareness are often left to connect foundational concepts to prescribing decisions on their own (Norman, 2009; Hartjes et al., 2024). This gap is consequential: safe prescribing depends on pharmacologic reasoning, and failures to connect pharmacology to clinical decision-making can lead to patient harm and broader public health consequences (Wiernik, 2015; Brinkman et al., 2018; Fasinu and Wilborn, 2024; Guilding et al., 2026).

This challenge often persists in the clinical years, where prescribing is frequently learned through pattern recognition, treatment algorithms, and workplace experience (Fasinu and Wilborn, 2024; Hartjes et al., 2024). While these approaches may support performance in familiar scenarios, they can limit reasoning when patients present atypically or require therapeutic adjustment. Evidence from integrated and problem-based curricula suggests that learners benefit when stronger conceptual scaffolding is provided in the early stages of healthcare training (e.g., preclerkship) for a deeper understanding of pharmacologic principles to confidently apply knowledge in prescribing contexts (Norman, 2009; Gillespie et al., 2022; Fasinu and Wilborn, 2024; Nicolaou et al., 2024). This is, at least in part, because overly contextualized knowledge can limit transfer of conceptual understanding across several scientific fields (National Academies of Sciences, Engineering, and Medicine, 2000).

Safe prescribing requires linking drug mechanisms to clinical decisions, which we refer to as mechanism-to-management reasoning (Bloch et al., 2025).

Mechanism-to-management reasoning refers to the ability to apply foundational pharmacological knowledge—including drug mechanisms, pharmacokinetics, pharmacodynamics, adverse effects, and patient-specific factors—to therapeutic decision-making. For example, a learner demonstrating mechanism-to-management reasoning would be able to explain how impaired renal function alters drug disposition and use that understanding to justify dose selection, monitoring strategies, or alternative therapies. This form of reasoning connects conceptual understanding to prescribing actions and supports adaptation when patients present with complexity, uncertainty, or atypical clinical circumstances.

A more coherent approach is to use backward design, aligning performance-focused learning outcomes with instruction and assessment that support the development of competencies required for prescribing activities such as EPA 4. Building these learning outcomes on the core concepts of the discipline is important because deeper conceptual understanding aids the transfer of understanding from one context to another (National Academies of Sciences, Engineering, and Medicine, 2000; Doherty et al., 2023; Franovic et al., 2023). In pharmacology, these outcomes can be grounded in established disciplinary frameworks, including International Union of Basic and Clinical Pharmacology (IUPHAR) Core Concepts (White et al., 2023; American Society for Pharmacology and Experimental Therapeutics, 2024; Guilding et al., 2024), which underscore principles such as drug efficacy, dose–response relationship, drug distribution, and inter-individual variability in response, and mechanisms of toxicity.

Endeavors like those frame foundational concepts within a defined prescribing competency, such as EPA 4, ensuring that pharmacology education moves beyond rote memorization to explicitly support clinical decision-making.

**FIGURE 1:**
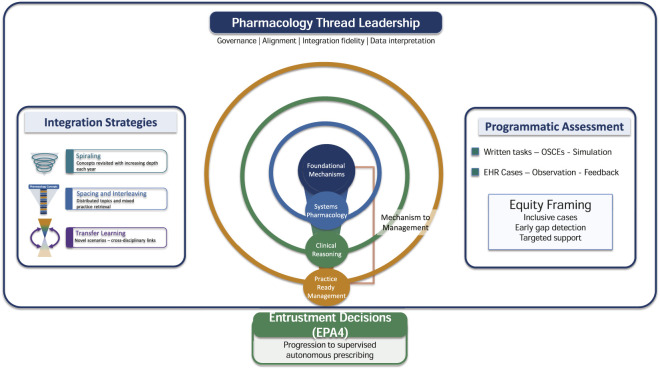
Conceptual model for pharmacology thread leadership in competency-based medical education. The figure illustrates how pharmacology thread leadership coordinates cognitive integration strategies, programmatic assessment, and equity-focused educational practices to support prescribing competence and Entrustable Professional Activity 4 (EPA 4). Integration strategies, including spiraling, spacing, interleaving, and transfer learning, reinforce pharmacologic reasoning across time and contexts. These approaches support the progression from foundational mechanisms to systems pharmacology, clinical reasoning, and practice-ready medication management through mechanism-to-management reasoning. Programmatic assessment incorporates multiple assessment modalities, including written tasks, Objective Structured Clinical Examinations (OSCEs), simulations, electronic health record (EHR)-based cases, workplace observation, and feedback. Equity framing emphasizes inclusive case design, early identification of learning gaps, and targeted learner support. Together, these elements contribute to well-supported entrustment decisions related to supervised autonomous prescribing within EPA 4.

To support this longitudinal development, we propose structuring pharmacology education through three evidence-based strategies (Figure 1): Spiraling, revisiting core concepts with a greater degree of depth and increasing clinical complexity; Spacing, reinforcing pharmacologic reasoning over time to support retention and application in clinical settings; and Interleaving, integrating contrasting drug classes, mechanisms, and patient scenarios to strengthen conceptual understanding and therefore therapeutic decision-making.

These strategies are well supported in the learning sciences and have demonstrated effectiveness in pharmacology education (Harden, 2000; Kerfoot et al., 2007; Rohrer, 2012). Spaced retrieval through cumulative assessments or longitudinal cases further strengthens retention and transfer of knowledge to clinical practice (Kerfoot et al., 2007). Interleaving across drug classes and clinical scenarios enhances learners’ ability to distinguish among therapeutic options and supports decision-making under uncertainty (Rohrer, 2012).

Positioning pharmacology as a longitudinal thread provides the structure needed to implement these strategies throughout undergraduate medical education, an approach supported by prior work demonstrating the effectiveness of spiraling, integrated pharmacology curricula that reinforce learning over time and across clinical contexts (Karpa and Vrana, 2013; Karpa et al., 2015). This includes both vertical (across training phases) and horizontal (with related foundational biomedical sciences disciplines such as physiology, pathology, and biochemistry) integration. Understanding the role of drug targets in physiology and pathology enables learners to predict therapeutic and adverse effects and to adapt treatment to patient-specific contexts. When these connections are revisited across time and settings, learners are better able to apply pharmacological reasoning in authentic prescribing situations (Harden, 2000; Youm et al., 2024).

The proposed model is informed by complementary educational theories and implementation frameworks that together support the longitudinal development of prescribing competence. Educational theories, including transfer of learning, situated learning, and self-regulated learning, provide the conceptual foundation for helping learners progressively apply pharmacological knowledge across increasingly authentic clinical contexts while assuming greater responsibility for their own learning. Competency-based medical education provides the developmental framework for progression toward prescribing competence, while entrustment theory informs how evidence from multiple assessment sources can support decisions about readiness for supervised prescribing activities (Frank et al., 2010; Katyal and Krishnan, 2025). Programmatic assessment theory holds that meaningful judgments about competence should be based on the synthesis of multiple low- and high-stakes assessment data collected over time, rather than on isolated examinations (Katyal and Krishnan, 2025). These principles are operationalized through evidence-based educational frameworks, including backward design, competency-based medical education, programmatic assessment, and entrustable professional activities (Ten Cate and Hennus, 2024). Together, these theories and frameworks support the role of pharmacology thread leadership by providing a coherent structure for aligning curriculum, assessment, learner development, and decisions about readiness for prescribing practice.

## 3 From content delivery to governance: establishing pharmacology thread leadership

The transition to CBME requires a fundamental shift in how pharmacology is led, coordinated, assessed, taught, and evaluated within integrated curricula. Pharmacology educators have traditionally functioned as distributed content experts, often teaching effectively within individual courses but with limited authority over curricular coherence, developmental progression, or assessment strategy beyond their own discipline. Under these conditions, pharmacology may be taught well locally but remains weakly coordinated at the program level. This fragmentation is incompatible with the longitudinal, integrated development required for safe prescribing, a well-recognized global patient safety priority (Heaton et al., 2008; Maxwell et al., 2015; World Health Organization, 2017).

Because prescribing competence develops over time, across settings, and depends on integrated reasoning, it requires a governance structure capable of sustaining coherence across the curriculum. We define pharmacology thread leadership as a program-level role responsible for the longitudinal integration, developmental sequencing, alignment, and validity of the system of assessment, evaluation, and quality oversight of pharmacology within an integrated curriculum. This role, often called a thread leader, is not an administrative convenience but a solution to a core educational design problem.

Importantly, pharmacology thread leadership is not intended to function in isolation. Prescribing education and assessment are inherently collaborative activities that benefit from the expertise of pharmacists, physicians, nurse prescribers, assessment specialists, and other healthcare professionals. Within this model, pharmacology thread leaders serve as coordinators and stewards of pharmacological reasoning while working in partnership with colleagues who contribute complementary clinical, educational, and professional perspectives.

**FIGURE 2:**
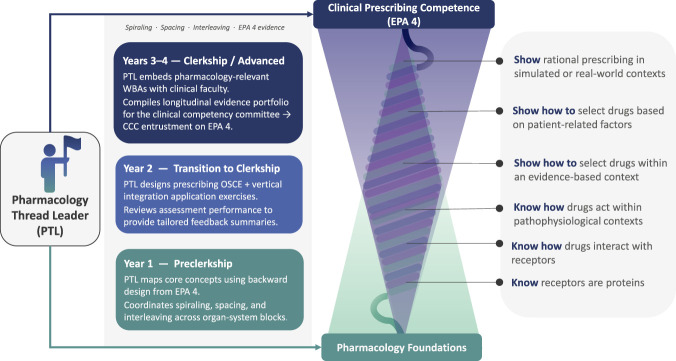
Example of pharmacology thread leadership in practice across a 4-year medical curriculum. This figure operationalizes the proposed model by illustrating how a Pharmacology Thread Leader (PTL) integrates cognitive science, programmatic assessment, and entrustment-based frameworks within a single accountable governance role. The vertical axis maps developmental progression along Miller’s pyramid, beginning with foundational pharmacological knowledge and culminating in clinical prescribing competence (EPA 4). At each phase, the PTL performs a specific, observable function: in Year 1, mapping core concepts through backward design and coordinating spiraling, spacing, and interleaving across organ-system blocks; in Year 2, designing prescribing OSCEs with vertical integration and delivering tailored feedback summaries; and in Years 3 and 4, embedding pharmacology-relevant workplace-based assessments (WBA) and compiling longitudinal evidence portfolios that inform clinical competency committee entrustment decisions.

The value of pharmacology thread leadership lies not simply in coordinating existing educational activities, but in serving as the mechanism through which previously disconnected elements become part of a coherent developmental system. Learning science principles such as spiraling, spacing, and interleaving; competency-based frameworks such as EPA 4; and programmatic assessment strategies have each been described independently in the literature (Van Der Vleuten et al., 2012; 2015; Torre et al., 2022; Ten Cate and Hennus, 2024). However, implementation often remains fragmented because responsibility for connecting these components across courses, training phases, and assessment systems is diffuse. Pharmacology thread leadership provides an organizational structure through which these educational approaches can be intentionally aligned, monitored, and continuously improved. In this model, the thread leader functions as a steward of continuity, ensuring that pharmacological reasoning is progressively developed, assessed, and incorporated into decisions about readiness for prescribing practice. Figure 2 illustrates a proposed model on the operationalization of the Pharmacology Thread Leader roles in a medical curriculum.

Pharmacology thread leaders should have responsibility for mapping core concepts across the curriculum, aligning pharmacology assessments with prescribing-related Entrustable Professional Activities (EPAs), particularly EPA 4, and interpreting longitudinal performance data across classroom, simulation, and clinical settings (Ten Cate, 2013; Association of American Medical Colleges, 2014; Englander et al., 2016). International efforts, including the IUPHAR core concepts initiative (Guilding et al., 2024; 2026), reinforce the need to apply pharmacological knowledge across contexts to support clinical reasoning and safe prescribing (National Academies of Sciences, Engineering, and Medicine, 2000).

This role requires formal authority, including access to curricular and assessment data, participation in curriculum and assessment governance, and protected time. Without these supports, pharmacology may remain visible as content, but fails to translate into demonstrable competence (Heaton et al., 2008; Brinkman et al., 2018). Within this model, integration fidelity refers to the degree of alignment among pharmacology learning outcomes, instructional experiences, assessment activities, and expectations for prescribing practice across the curriculum. Rather than representing a validated measurement construct, integration fidelity is intended as a framework for monitoring curricular coherence over time. Indicators may include the extent to which pharmacology core concepts are represented across courses, the consistency between learning outcomes and assessment blueprints, the reinforcement of prescribing-related concepts across training phases, and the degree to which prescribing competencies are sampled in authentic clinical contexts.

Variability in pharmacology education has been associated with concerns regarding prescribing preparedness among medical students and recent graduates, underscoring the importance of coordinated oversight and longitudinal monitoring of prescribing competence (Heaton et al., 2008; Brinkman et al., 2017; 2018).

Effective governance also requires distributed collaboration with central accountability. Pharmacology thread leaders must work closely with other foundational science thread leaders, clinical faculty, assessment leaders, and curriculum committees while maintaining responsibility for integration. Initiatives such as the Prescribing Safety Assessment (PSA), developed by the British Pharmacological Society, demonstrate how coordinated, multi-stakeholder approaches can support prescribing competence at scale (Maxwell et al., 2015; Magavern et al., 2024; McLachlan et al., 2025).

Finally, sustainable implementation depends on institutional recognition of this role. Integrating pharmacology across curricula, aligning assessments, and monitoring outcomes require substantial expertise and effort. Institutions must provide faculty development, administrative support, and data infrastructure to ensure that pharmacology leadership is not symbolic but operational (Fasinu and Wilborn, 2024; Guilding et al., 2026).

## 4 Programmatic assessment architecture for prescribing competence

If prescribing competence develops over time, across contexts, and through a combination of mechanistic and clinical reasoning, it cannot be determined by a single high-stakes examination. Programmatic assessment provides a system-level approach in which multiple low-stakes observations are combined to inform high-stakes decisions about readiness for practice (Hauer et al., 2015).

This approach rests on three principles: longitudinal sampling across time and settings, triangulation across assessment methods, and deliberate separation of formative feedback from summative decision-making (Van Der Vleuten et al., 2012; 2015). Rather than relying on isolated tests, learners build a body of evidence through repeated observations, which is then reviewed by a competency committee to determine readiness for prescribing responsibilities.

Programmatic assessment must span Miller’s pyramid, encompassing the layers of knowledge (knows), competence (knows how), performance (shows how), and action (does) (Miller, 1990). It should incorporate written assessments of pharmacologic reasoning, prescribing OSCEs, simulation, electronic health record (EHR)-based tasks, and workplace-based assessments supported by multi-source feedback (Gillespie et al., 2022; Chin et al., 2023). Each method contributes complementary evidence, strengthening the validity of decisions.

Importantly, the proposed model is not intended to create an additional checklist of prescribing milestones. A common criticism of competency-based medical education is that complex professional activities may become fragmented into discrete tasks that are documented but not meaningfully integrated. Pharmacology thread leadership can help mitigate this risk by emphasizing developmental trajectories rather than isolated assessment events. Decisions regarding readiness for prescribing should be informed by multiple observations collected across settings, assessors, and levels of complexity, with attention to patterns of performance over time. In this way, prescribing competence is viewed as an evolving capability that emerges through repeated application of pharmacological reasoning in authentic clinical contexts rather than the completion of individual assessment requirements. Accordingly, EPA four should be interpreted as a longitudinal professional activity supported by accumulating evidence of competence rather than as a single assessment endpoint or discrete curricular milestone.

Program quality depends on monitoring the fidelity of integration across the assessment system.

For example, curricular mapping may be used to determine whether pharmacology core concepts are introduced, reinforced, and applied across multiple phases of training. Assessment blueprints can be reviewed to evaluate whether prescribing-related competencies are sampled consistently across written examinations, simulations, and workplace-based assessments. Longitudinal learner performance data may identify areas where pharmacological reasoning improves, plateaus, or declines over time, while progression metrics can be used to examine whether increasing levels of prescribing responsibility are supported by corresponding evidence of competence. Together, these approaches provide practical indicators for monitoring integration fidelity and informing continuous curriculum improvement. Inputs include coverage of prescribing tasks, spacing of pharmacology reinforcement, and representation of diverse clinical contexts. Processes include sampling density, assessor training and participation, and feedback quality. Outputs include decision reliability, progression patterns, and prescribing safety indicators (Van Melle et al., 2019; Cooper and Holmboe, 2025).

Although the Prescribing Safety Assessment (PSA) provides an important example of national coordination and benchmarking of prescribing competence, it should not be viewed as a complete model of programmatic assessment. High-stakes examinations can offer valuable information regarding learner performance at specific points in training, but they capture only a portion of the evidence needed to support judgments about readiness for prescribing practice. Consistent with principles of programmatic assessment, decisions regarding prescribing competence should be informed by multiple sources of evidence collected across time, settings, and assessment modalities, including workplace-based observations, simulations, prescribing exercises, and clinical performance data.

Authenticity is critical. Simulation-based tasks and EHR-integrated cases can approximate clinical complexity while reducing variability across settings (Arcoraci et al., 2019; Miller et al., 2021; Firman et al., 2025). Emerging generative artificial intelligence tools may further support standardized practice environments. However, they must not replace work-based assessment but instead complement it to preserve clinical authenticity (Izquierdo-Condoy et al., 2025; Guilding et al., 2026).

Ultimately, decisions about readiness for practice must be well-supported by multiple sources of evidence and guided by transparent decision rules aligned with established entrustment frameworks (Hauer et al., 2015; De Graaf et al., 2021; Lonsdale et al., 2026). Within the proposed model, assessments such as the PSA are best viewed as a single data point within a larger longitudinal system of evidence rather than as standalone indicators of prescribing readiness. Without explicit pharmacology leadership in designing and interpreting this system, discipline-based assessment risks producing data without improving prescribing safety (Torre et al., 2022; Guilding et al., 2026).

## 5 Enablers: equity, faculty development, interprofessional collaboration and system sustainability

Successful implementation of pharmacology-informed assessment systems depends on four key elements: equity, faculty development, interprofessional collaboration, and sustainability.

Equity requires deliberate attention to differences in learners’ backgrounds, experiences, and opportunities. Assessment systems should incorporate inclusive case design, represent diverse patient populations, and enable longitudinal tracking of pharmacology-related competencies. Equitable assessment practices include the use of clear performance criteria, rater training and calibration, multiple sources of evidence, and systematic review of outcomes to identify potential bias. Although variability in educational background may influence mastery of pharmacologic concepts (Woolf et al., 2013), educators must distinguish between true performance differences and assessment-related inequities and address disparities early in the learning process.

Faculty development is equally critical. Effective pharmacology thread leadership requires competencies that extend beyond disciplinary expertise. In many institutions, the initial implementation of pharmacology thread leadership may be most feasible through enhancement of existing educational leadership roles rather than creation of new positions. Pharmacology educators may need training in assessment design, assessment literacy, curriculum leadership, data interpretation, quality improvement, and collaborative decision-making. Programmatic assessment requires educators to synthesize longitudinal performance data, provide meaningful feedback, participate in competency committee discussions, and contribute to high-stakes decisions regarding learner progression (Schuwirth and van der Vleuten, 2011). These capabilities should not be assumed solely on the basis of pharmacology expertise and may entail targeted faculty development, mentoring, and opportunities to participate in curriculum governance and assessment activities.

Many pharmacology educators may not receive formal preparation in these areas during their scientific or clinical training. Consequently, institutions seeking to implement this model should consider targeted faculty development opportunities that prepare pharmacology educators to engage effectively in curriculum planning, assessment oversight, and educational decision-making. Developing these capabilities will be essential if pharmacology thread leaders are to contribute meaningfully to longitudinal assessment systems and institutional educational reform. Equally important, institutions must provide thread leaders with meaningful participation in curriculum committees and assessment governance structures if they are expected to influence educational practice beyond their own courses. In other words, effective implementation is directly related to institutional commitment.

The implementation process should occur within an interprofessional framework. Safe and effective prescribing is a shared responsibility involving physicians, pharmacists, nurse practitioners, physician associates/assistants, nurses, and other healthcare professionals. Because prescribing is an interprofessional activity, implementation of pharmacology thread leadership should occur within collaborative educational structures (Collins and McLain, 2021). Incorporating these perspectives can strengthen the design and evaluation of prescribing curricula while ensuring that learners encounter prescribing as a shared professional responsibility rather than a discipline-specific task. Consequently, assessment systems should benefit from the perspectives of multiple professions when designing assessment strategies, interpreting learner performance, and identifying opportunities for improvement.

Interprofessional collaboration can strengthen the authenticity, relevance, and validity of pharmacology-related assessments while reinforcing team-based approaches to medication management.

## 6 A transferable model for foundational sciences

Although this framework is centered on pharmacology, it addresses a broader challenge in CBME: the perceived marginalization of foundational sciences in assessment systems (Norman, 2009; Finnerty et al., 2010; Gruppen et al., 2016; Quesnelle et al., 2021). Disciplines such as physiology, pathology, and microbiology face similar issues of fragmentation, limited alignment with clinical practice, and reduced visibility in decisions about readiness for practice (Norman, 2009).

Adaptation requires discipline-specific approaches to authentic assessment. For example, pathology may emphasize diagnostic reasoning, while microbiology may focus on infection management. However, the underlying principle remains consistent: foundational sciences must be explicitly linked to clinical decision-making to remain relevant within CBME (Finnerty et al., 2010; Frank et al., 2010; Englander et al., 2016). This model should be applied as a guiding structure rather than a rigid template, allowing for local adaptation while maintaining coherence and accountability.

The proposed model offers three transferable components: program-level leadership structures, monitoring of integration fidelity, and alignment of assessment with clinical competencies. Together, these elements position foundational sciences as drivers of clinical reasoning rather than repositories of isolated knowledge. Sustaining this approach across disciplines will require shared governance models in which foundational science thread leaders collectively support curricular alignment, assessment coherence, and readiness decisions rather than operating through isolated disciplinary oversight.

## 7 Research agenda and call to action

The novelty of this Perspective lies less in the individual educational components it discusses than in the governance structure that connects them. Previous work has established the importance of pharmacology core concepts, therapeutic reasoning, prescribing assessment, and competency-based curricular design (Fasinu and Wilborn, 2024; Guilding et al., 2024; 2026; McLachlan et al., 2025; Lonsdale et al., 2026). However, these efforts largely focus on educational content, competencies, assessment approaches, or curricular recommendations. We argue that sustainable improvement in prescribing competence also requires explicit ownership of the longitudinal assessment system itself. By positioning pharmacology thread leaders as accountable stewards of integration, assessment coherence, and prescribing outcomes, we offer a complementary organizational framework that may help translate existing educational innovations into durable program-level practice.

Despite widespread adoption of CBME (Udemans et al., 2018; Jacob, 2019; De Graaf et al., 2021; Frank et al., 2024), evidence linking educational reform to improved patient outcomes remains limited (Hamza et al., 2023). Pharmacology educators are uniquely positioned to address this gap by focusing on prescribing competence as a measurable and clinically meaningful outcome.

Key priorities include evaluating the impact of programmatic assessment on prescribing safety, identifying effective models across institutions, and developing standardized approaches for benchmarking competence (Chin et al., 2023; Donker et al., 2025). Multi-institutional, mixed-methods research is well-suited to determine what works, for whom, and under what conditions (Cook et al., 2008).

To translate this agenda into action, we propose three commitments. First, pharmacology educators should adopt or adapt shared EPA-based frameworks for assessing prescribing competence (Mejicano et al., 2017). Second, they should assume leadership of assessment systems and use longitudinal data to support benchmarking and continuous improvement (thread leader) (Guilding et al., 2026; Linsenmeyer and Gusic, 2026). Third, they should invest in faculty development to build expertise in assessment, integration, and educational leadership (Cho et al., 2025). These steps will help ensure that CBME fulfills its promise of improving patient care rather than retaining an organizational reform.

## 8 Conclusion

The shift to competency-based medical education requires more than curricular change; it requires a redefinition of disciplinary roles. Pharmacology educators must move beyond content delivery to lead the assessment of prescribing competence, ensuring that readiness for practice is demonstrated through integrated, longitudinal evidence. By aligning cognitive integration, governance, and discipline-based assessment, pharmacology can serve as a model for foundational sciences seeking to improve clinical outcomes. This shift is essential for advancing patient safety, educational equity, and the credibility of competency-based medical education.

## Data Availability

The original contributions presented in the study are included in the article/supplementary material, further inquiries can be directed to the corresponding author.
